# Brain alterations in adolescents with first-episode depression who have experienced adverse events: evidence from resting-state functional magnetic resonance imaging

**DOI:** 10.3389/fpsyt.2024.1358770

**Published:** 2024-04-09

**Authors:** Xiaodi Xia, Jinxiang Tang, Yadong Peng, Ying Liu, Yingying Chen, Meng Yuan, Renqiang Yu, Xiao Hou, Yixiao Fu

**Affiliations:** ^1^ Department of Psychiatry, The First Affiliated Hospital of Chongqing Medical University, Chongqing, China; ^2^ Department of Radiology, The First Affiliated Hospital of Chongqing Medical University, Chongqing, China; ^3^ Department of Clinical Medicine, Chongqing Medical and Pharmaceutical College, Chongqing, China

**Keywords:** major depressive disorder, childhood trauma, negative life events, resting-state functional magnetic resonance imaging, adolescents

## Abstract

**Introduction:**

Adverse life events constitute primary risk factors for major depressive disorder (MDD), influencing brain function and structure. Adolescents, with their brains undergoing continuous development, are particularly susceptible to enduring impacts of adverse events.

**Methods:**

We investigated differences and correlations among childhood trauma, negative life events, and alterations of brain function in adolescents with first-episode MDD. The study included 23 patients with MDD and 19 healthy controls, aged 10–19 years. All participants underwent resting-state functional magnetic resonance imaging and were assessed using the beck depression inventory, childhood trauma questionnaire, and adolescent self-rating life events checklist.

**Results:**

Compared with healthy controls, participants with first-episode MDD were more likely to have experienced emotional abuse, physical neglect, interpersonal relationship problems, and learning stress (all *p’* < 0.05). These adverse life events were significantly correlated with alterations in brain functions (all *p* < 0.05).

**Discussion:**

This study contributes novel evidence on the underlying process between adverse life events, brain function, and depression, emphasizing the significant neurophysiological impact of environmental factors.

## 1 Introduction

Major depressive disorder (MDD) is characterized by an inability to feel joy or continuous emotions of despair (1). In China, MDD is the second most common cause of disability (2). Adolescents with MDD have symptoms that are more intense, last longer, and have higher recurrence rates than adults (3). Moreover, nearly half of all mental disorders begin during adolescence (4), emphasizing the importance of investigating the pathogenesis of first-episode MDD, as well as prevention and early intervention.

Adolescence is crucial for psychological growth and is affected by negative events in families, schools, and physical and psychological aspects (5). These events can directly impact depressive emotions and indirectly influence the emotions by affecting ruminant thinking (6), particularly in individuals with disruptions to default mode network functional connectivity (7). However, the precise neural mechanisms underlying these changes remain unclear.

Studies suggest that adults with MDD may have experienced early trauma and negative life events in their early life (8–10). These individuals display cognitive and emotional impairments associated with dysfunction in the resting-state functional connectivity (rs-FC) of the amygdala, the anterior insula (11), the hippocampus, and the temporal cortex.

The early-childhood stage of brain development renders the brain susceptible to adverse life events, but limited research has explored the effects of these stress on rs-FC among adolescents with first-episode MDD. Therefore, we aimed to investigate the differences in trauma, negative life events, and alterations of brain function between adolescents with first-episode MDD and healthy controls and analyze their correlations.

## 2 Materials and methods

### 2.1 Study design and participants

We utilized a case-control design in this study, recruiting 23 patients from the Psychiatry Department of the First Affiliated Hospital of Chongqing Medical Hospital and 19 healthy controls from local schools between May 2018 and August 2019. All participants were right-handed and of native Chinese Han ethnicity. Two licensed psychiatrists diagnosed the patients using the Structured Clinical Interview for the Diagnostic and Statistical Manual of Mental Disorders, Fifth Edition (DSM-V) Patient Edition for MDD. Inclusion criteria encompassed an age range of 10–19 years, a diagnosis of first-episode MDD without significant recovery, and no current use of antidepressants. Exclusion criteria included bipolar disorder, schizophrenia, any other psychotic disorder, severe medical conditions such as cardiac or organic brain disease, and contraindications to magnetic resonance imaging (MRI). Healthy controls adhered to the same exclusion criteria. This study adhered to the Declaration of Helsinki and was approved by the Medical Research Ethics Committee of the First Affiliated Hospital of Chongqing Medical University (Approval No. 2023-058-02). All adolescent participants and their guardians signed a written informed consent form after receiving a complete description of the study.

### 2.2 Measures

All participants underwent a comprehensive clinical assessment including general information, childhood trauma experience, and adverse life events. We used the beck depression inventory (BDI) (12), the childhood trauma questionnaire (CTQ) (13), and the adolescent self-rating life events checklist (ASLEC) (14) to assess severity of depression, experiences of physical, sexual, and emotional abuse, as well as physical and emotional neglect, and negative life events. The Chinese editions of both the CTQ and ASLEC have demonstrated reliability and validity in adolescents (15, 16).

### 2.3 Imaging data acquisition and pre-processing

All MRI data were obtained using a Signa HDxt 3.0-T MRI machine (GE Healthcare, Chicago, IL) with an eight-channel phased array head coil at the Department of Radiology of the First Affiliated Hospital of Chongqing Medical Hospital. During the resting-state functional MRI (rs-fMRI) scanning, participants were instructed to close their eyes and avoid thinking. The rs-fMRI data were acquired using a gradient-echo-echo-planar imaging sequence with specific parameters: time repetition/time echo = 2000/40 ms, flip angle = 90°, field of view = 100 × 100 mm, matrix = 64 × 64, slice thickness/slice spacing = 4.0/4.0 mm. High-resolution anatomical T1-weighted destructive gradient recall images were acquired, covering the whole brain, using the following parameters: time repetition/time echo = 8348/3.27 ms, flip angle = 12°, field of view = 75 × 75 mm, matrix = 256 × 256, slice thickness/slice spacing = 1.0/1.0 mm. To ensure the absence of structural abnormalities, two experienced neuroradiologists thoroughly examined all images.

RESTplus 1.25 (17), based on Statistical Parametric Mapping 12 in MATLAB 2013b (MathWorks, Natick, MA), was used for image data preprocessing. The main steps included: (1) removing the first 10 volumes to address scanner instability, (2) slice timing and head motion correction, ensuring displacement of participants < 3 mm/3°, (3) excluding non-neuronal sources, including white matter, cerebrospinal fluid, and 24-parameter head motion profile signals, (4) performing spatially normalized corrected imaging at a resolution of 3 mm × 3 mm × 3 mm, (5) calculating regional homogeneity (ReHo) and using z-standardization to a normal distribution, (6) smoothing the data using a Gaussian kernel of 6-mm full width and half maximum, (7) removing linear trend. We chose different zReHo values between patients with MDD and healthy controls as a region of interest (ROI), used the seed-based functional connectivity (FC) method to assess the abnormalities, and used Fisher’s z transformation to enhance normality.

### 2.4 Statistical analysis

Demographic data and clinical characteristics were compared using independent-sample t-tests for normal variables, the Mann–Whitney U-test for non-normal variables, and the chi-squared test for sex in SPSS 23.0 (IBM, Armonk, NY). Statistical significance was defined as *p* < 0.05 for all two-tailed tests. A two-sample t-test was used to compare ReHo between the MDD and healthy control groups, with an uncorrected significance level of *p* < 0.05. We used a one-sample t-test using false discovery rate (FDR) correction with cluster-level multiple comparison correlation of significance level of *p* < 0.05 to illustrate the spatial distribution of within-group FC for the MDD and healthy control groups. Following this, we conducted a two-sample t-test to determine whether there were any significant variations in whole-brain FC within the ROI between the two groups. In this part, the Gaussian random field theory was used for cluster-level multiple corrections, with a threshold of Voxel-level *p* < 0.05 and Cluster-level *p*-value < 0.05. The mean ReHo and FC values of abnormal brain regions were extracted for examining the correlations between the brain functional values and clinical characteristics, using Spearman’s correlation analysis. The *p*-values have been adjusted to Benjamini–Hochberg *p'*-values to control the FDR when necessary. Sex, age, and years of education were considered as nuisance factors.

## 3 Results

### 3.1 Demographic and clinical characteristics

In total, 42 participants were selected, of whom 23 were patients with MDD and 19 were healthy controls. The demographic and clinical data of all participants are presented in **Table 1**. Five patients with MDD were unable to complete the ASLEC and BDI.

**Table 1:** Demographic and clinical characteristics of study participants.

	MDD (n = 23)	HC (n = 19)		
	Mean (SD)		*p*	*p’*
Age (years)	16.62 (1.76)	15.00 (2.94)	0.112	0.173
Sex (male/female)	23 (7/16)	19 (10/9)	0.145#	0.193
Education (years)	10.26 (1.76)	9.00 (2.94)	0.112	0.173
CTQ-EA	10.65 (3.28)	8.26 (1.91)	0.006	0.019
CTQ-PA	6.96 (2.21)	6.05 (1.18)	0.119*	0.173
CTQ-SA	11.30 (1.64)	10.16 (0.96)	0.023*	0.053
CTQ-EN	16.57 (3.45)	14.84 (4.30)	0.089*	0.173
CTQ-PN	8.78 (3.40)	6.53 (1.22)	0.009*	0.024
CTQ-T	54.39 (9.82)	45.84 (5.88)	0.001	0.004
	n=18	n=19		
ASLEC-IR	15.67 (5.50)	8.11 (5.71)	0.000	0.000
ASLEC-LS	15.56 (5.00)	8.26 (4.40)	0.000	0.000
ASLEC-P	16.44 (6.49)	14.00 (11.37)	0.426	0.454
ASLEC-L	7.67 (3.77)	5.74 (4.75)	0.181	0.223
ASLEC-HA	8.78 (4.12)	7.21 (6.16)	0.512*	0.512
ASLEC-O	10.33 (3.57)	8.47 (5.88)	0.251	0.287
BDI	15.17 (5.09)	5.74 (5.50)	0.000*	0.000

ASLEC, adolescent self-rating life events checklist; BDI, beck depression inventory; CTQ, childhood trauma questionnaire; EA, emotional abuse; EN, emotional neglect; HA, health and adaptation problems; HC, healthy control; IR, interpersonal relationships; L, loss of relatives, friends, and property; LS, learning stress; MDD, major depressive disorder; O, others; P, punishment; PA, physical abuse; PN, physical neglect; SA, sexual abuse; SD, standard deviation; T, total score.

*: Mann–Whitney U test.

#: chi-squared test.

*p’*: *p* values after false discovery rate correction.

### 3.2 Group comparisons

Age, sex, and years of education did not significantly differ between the MDD and healthy control groups. Adolescents with MDD were much more likely than healthy controls to report experiencing emotional abuse or physical neglect, and their CTQ total score was also significantly higher (all *p’* < 0.05). Additionally, compared to the healthy controls, adolescents with MDD exhibited a greater prevalence of interpersonal relationship and learning stress problems (all *p’* < 0.001). No differences were observed between the two groups regarding physical and sexual abuse; emotional neglect; punishment; loss of relatives, friends, and property; health and adaptation problems; or others (all *p’* > 0.05).

### 3.3 Comparison of ReHo between groups

**Figure 1:**
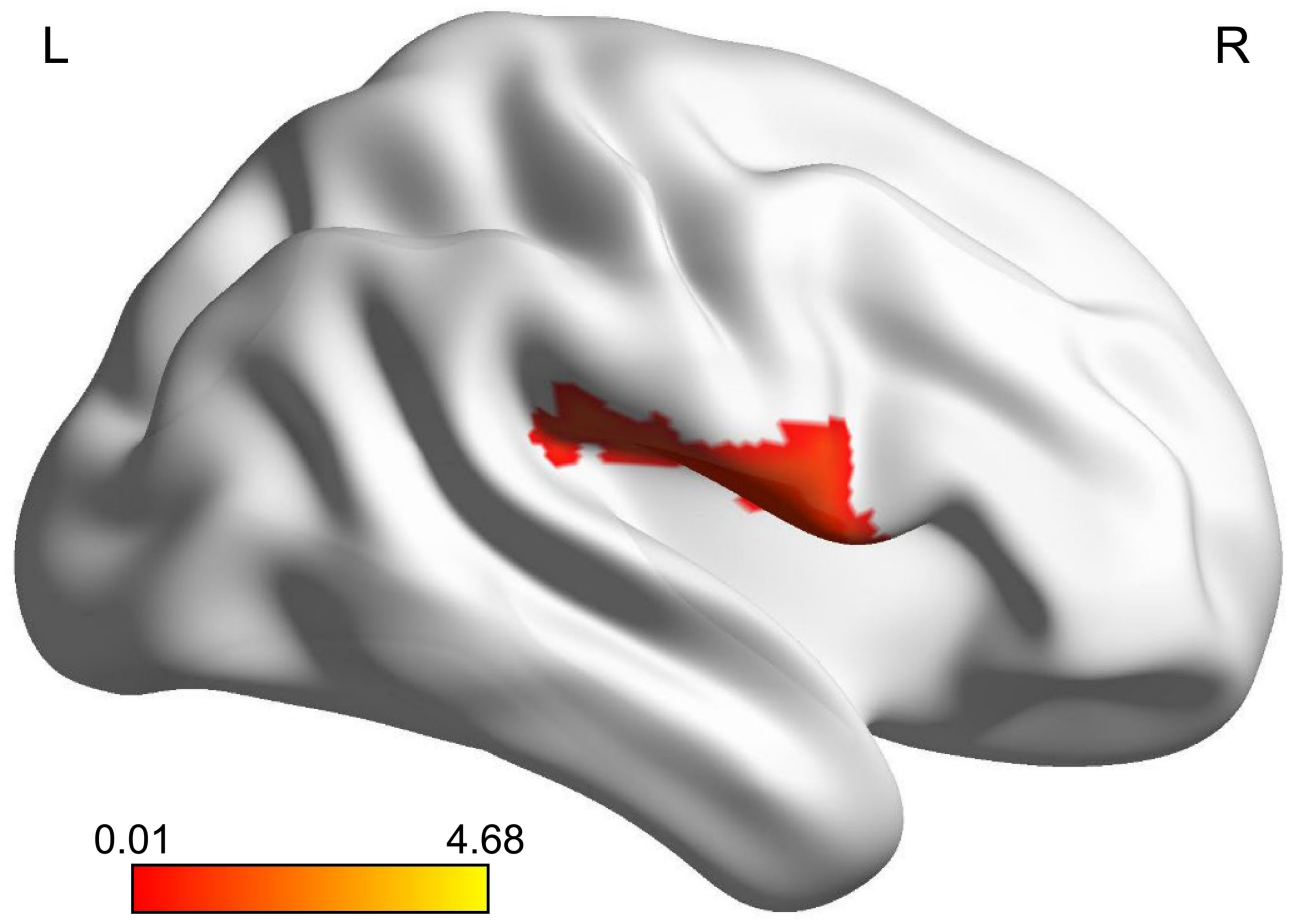
Brain region where adolescents with MDD had significantly more activity than healthy controls. The highlighted region was located on the most significantly different voxel of the right Rolandic operculum (X, Y, Z = 51, -6, 12, T = 4.678, P_FDR_ < 0.05). The color bar represents the T value of zReHo. zReHo, z-standardized regional homogeneity. MDD, major depressive disorder.

The zReHo of the right Rolandic operculum (ROL.R) was significantly higher in the MDD group than in the healthy control group (*p_FDR_* < 0.05) (**Table 2**; **Figure 1**).

**Table 2:** Comparison of z-standardized regional homogeneity.

Brain region	MNI coordinates x, y, z	Volume (voxels)	T value
Right Rolandic operculum	51, -6, 12	1344	4.678

MNI, montreal neurological institute. (P_FDR_ < 0.05, Cluster size > 1344).

### 3.4 Within-group FC analysis

**Figure 2:**
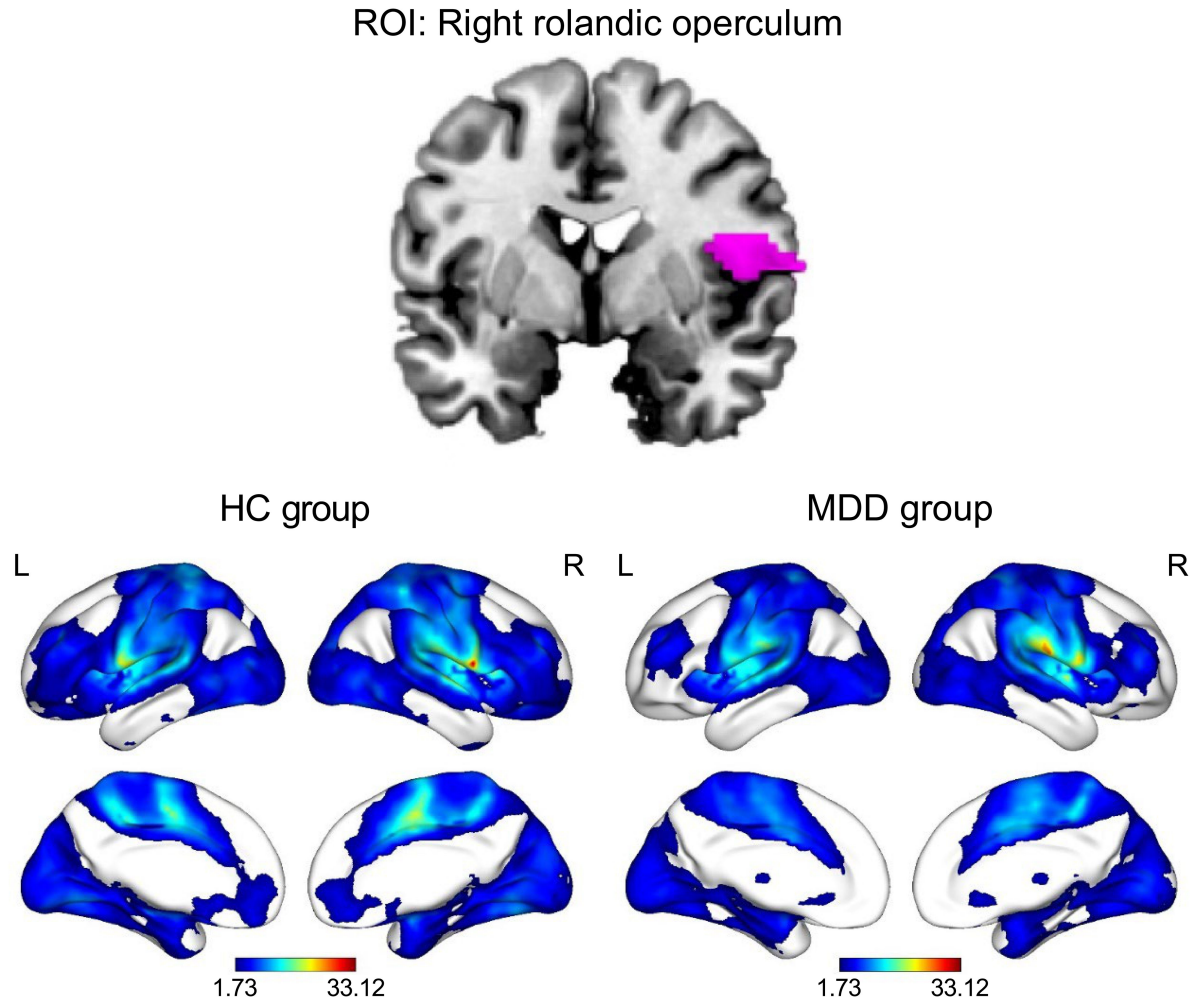
Functional connectivity patterns of the right Rolandic operculum in the HC and MDD groups (P_FDR_ < 0.05, cluster size > 10). The color bar represents the T value of functional connectivity. HC, healthy control; L, left; MDD, major depressive disorder; R, right; ROI, region of interest.

The results of the one-sample t-test revealed that the ROL.R with high FC values had a strong connection to the cingulate cortex. Furthermore, activities were observed in the temporal cortex, frontal cortex, caudate, angular gyrus, and parahippocampal gyrus (**Table 3**). The spatial distribution of FC in the MDD group showed similar patterns to those observed among healthy controls (**Table 4**; **Figure 2**).

**Table 3:** Functional connectivity of right Rolandic operculum for healthy controls.

Brain region	MNI coordinates x, y, z	Volume (voxels)	T value
Right Rolandic operculum	54, 3, 9	33471	33.123
Right posterior cingulate gyrus	9, -39, -18	12	2.962
Right inferior temporal	57, -3, -33	20	-2.360
Right middle frontal gyrus, orbital part	3, 66, -15	13	-2.593
Left parahippocampal gyrus	-33, -45, 0	16	-4.786
Left caudate nucleus	-18, 24, 12	28	-6.756
Right caudate nucleus	21, 27, 12	22	-5.305
Right precuneus	24, -45, 12	11	-4.736
Right superior frontal gyrus	27, 27, 60	1223	-6.918
Left angular gyrus	-39, -51, 24	24	-2.716
Left middle occipital gyrus	-48, -72, 39	111	-3.073
Right angular gyrus	45, -75, 42	78	-3.170

MNI, montreal neurological institute. (P_FDR_ < 0.05, Cluster size > 10).

**Table 4:** Functional connectivity of right Rolandic operculum for patients with major depressive disorder.

Brain region	MNI coordinates x, y, z	Volume (voxels)	T value
Right postcentral gyrus	60, -15, 18	28867	27.235
Right inferior temporal gyrus	63, -18, -27	132	-4.017
Left precuneus	-21, -48, 12	17	-4.640
Left inferior temporal gyrus	-63, -12, -27	30	-2.781
Right temporal: superior temporal gyrus	42, 18, -30	15	-2.344
Left superior frontal gyrus	-36, 18, 60	2161	-6.328
Right inferior frontal gyrus, orbital part	45, 39, -18	11	-2.122
Left middle frontal gyrus, orbital part	-30, 60, -6	13	-2.183
Left anterior cingulate and paracingulate gyri	0, 51, 12	12	-2.042
Left precuneus	-21, -54, 27	287	-3.205
Right angular gyrus	42, -78, 42	184	-3.858
Left angular gyrus	-39, -81, 39	208	-3.569

MNI, montreal neurological institute. (P_FDR_ < 0.05, Cluster size > 10).

### 3.5 Between-group FC differences

**Figure 3:**
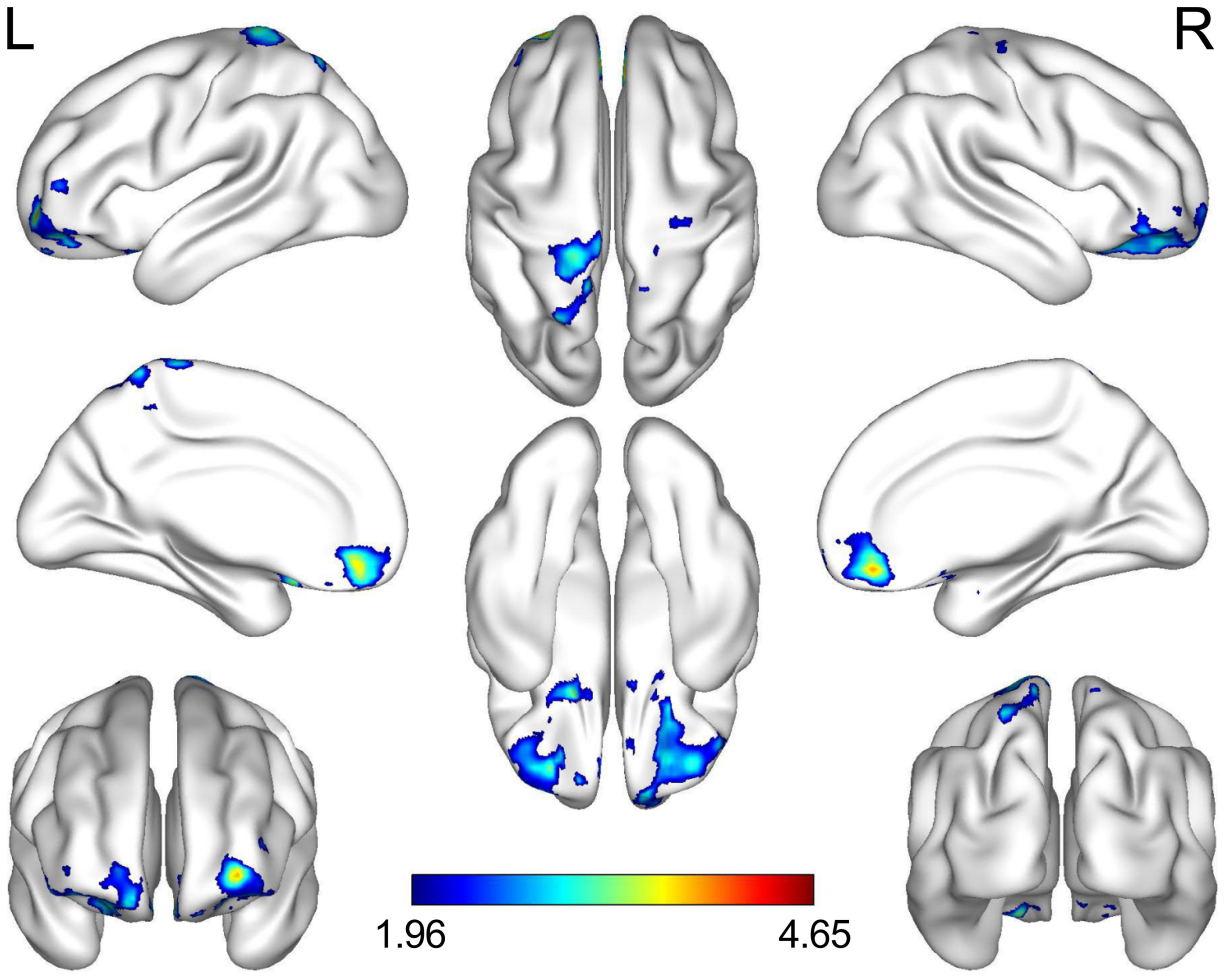
Brain regions where adolescents with MDD had less connectivity than the HCs. The highlighted regions were located on the most significantly different voxels of the right orbital part of the middle frontal and the left postcentral gyrus (X, Y, Z = 27, 36, -21, T = 4.646; X, Y, Z =-21, -39, 78, T = 4.122; voxel p < 0.05, cluster p < 0.05, false discovery rate corrected). The color bar represents the T value of functional connectivity. L, left; R, right; MDD, major depressive disorder; HC, healthy control.

Decreased FC between the ROL.R, the right orbital part of the middle frontal gyrus (ORBmid.R) and the left postcentral gyrus (PoCG.L) was observed more in the MDD group than in the healthy control group (**Table 5**; **Figure 3**).

**Table 5:** Functional connectivity differences between the major depressive disorder and healthy control groups with right Rolandic operculum as region of interest.

Brain region	MNI coordinates x, y, z	Volume (voxels)	T value
Right middle frontal gyrus, orbital part	27, 36, -21	1378	4.646
Left postcentral	-21, -39, 78	553	4.112

MNI, montreal neurological institute. (Voxel-level p < 0.05, Cluster-level p < 0.05, false discovery rate corrected, healthy controls > patients with major depressive disorder).

### 3.6 Correlation analyses

In the MDD group, the BDI score showed a positive correlation with the CTQ total score (*p* = 0.037, r = 0.524), physical neglect (*p* = 0.019, r = 0.579), punishment (*p* = 0.027, r = 0.61), and other (*p* = 0.003, r = 0.775). The increased ReHo was positively correlated with health and adaption problems (*p* = 0.004, r = 0.74). The decreased FC between ROL.R and PoCG.L was negatively correlated with sexual abuse (*p*= 0.039, r = -0.519).

In the HC group, the BDI score showed a positive correlation with emotional abuse (*p* = 0.004, r = 0.742). Additionally, each factor of the ASLEC showed a positive correlation with the BDI score (interpersonal relationships *p* = 0.000, r = 0.853; learning stress *p* = 0.001, r = 0.818; punishment *p* = 0.012, r = 0.67; loss of relatives, friend, and property *p* = 0.006, r = 0.715; health and adaption problems *p* = 0.023, r = 0.623; others *p* = 0.038, r = 0.579). Furthermore, the decreased ReHo was negatively correlated with emotional neglect (*p* = 0.028, r = -0.608).

**Figure 4:**
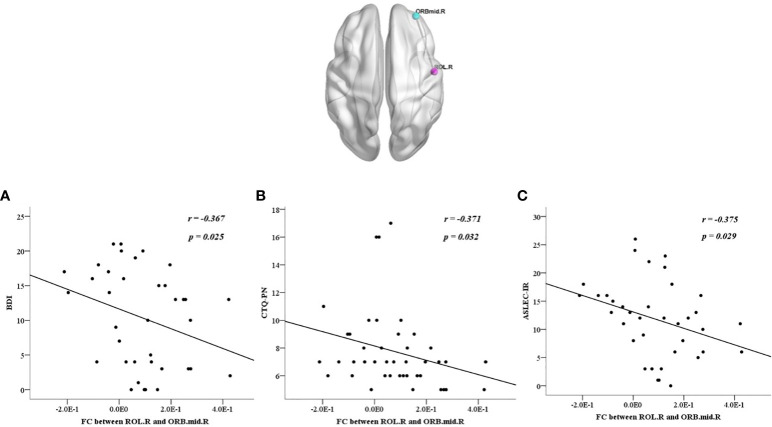
All participants showed negative correlations between the discrepant regions of functional connectivity in the right orbital part of the middle frontal gyrus and the right Rolandic operculum with BDI score **(A)**, CTQ-PN **(B)**, ASLEC, adolescent self-rating life events checklist; BDI, beck depression inventory; CTQ, childhood trauma questionnaire; FC, functional connectivity; ORBmid.R, right orbital part of the middle frontal gyrus; ROL.R, right Rolandic operculum.

**Figure 5:**
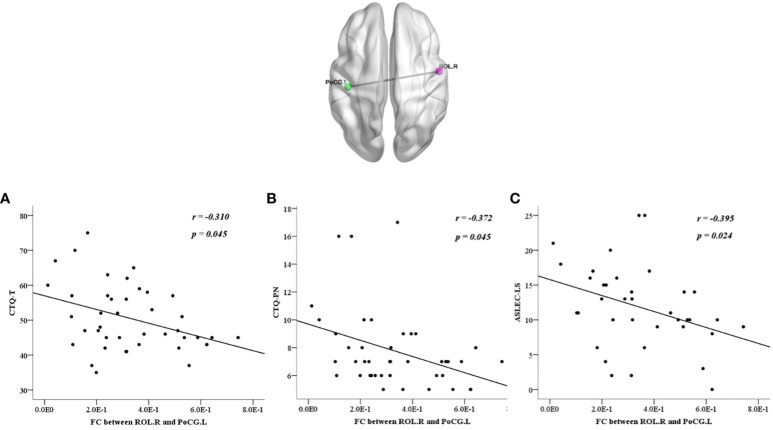
All participants showed negative correlations between the discrepant regions of functional connectivity in the left postcentral gyrus and the right Rolandic operculum with CTQ-T **(A)**, CTQ-PN **(B)**, and ASLEC-LS **(C)**. ASLEC, Adolescent Self-Rating Life Events Checklist; CTQ, Childhood Trauma Questionnaire; FC, functional connectivity; PoCG.L, left postcentral gyrus; ROL.R, right Rolandic operculum.

We also calculated the correlations between abnormalities and clinical characteristics in all participants, the BDI score was positively correlated with each factor of the CTQ and ASLEC (all *p* < 0.05), except health and adaptation problems (*p* > 0.05) (**Table 6**). The abnormal ReHo in the ROL.R was positively correlated with the BDI score, CTQ total score, emotional, physical, and sexual abuse, physical neglect, and interpersonal relationships (all *p* < 0.05). The abnormal FC between the ROL.R and ORBmid.R showed a negative correlation with the BDI score, physical neglect, and interpersonal relationship problems (all *p* < 0.05). The abnormalFC between the ROL.R and PoCG.L was negatively correlated with the CTQ total score, physical neglect, and learning stress problems (all *p* < 0.05) (**Figures 4**, **5**).

**Table 6:** Correlations between depression severity, childhood trauma and negative life events.

	CTQ-T	CTQ-EA	CTQ-PA	CTQ-SA	CTQ-EN	CTQ-PN	ASLEC-IR	ASLEC-LS	ASLEC-P	ASLEC-L	ASLEC-H	ASLEC-O
BDI	0.584**	0.559**	0.388*	0.398*	0.328*	0.558**	0.671**	0.732**	0.381*	0.407*	0.230	0.467**

ASLEC, adolescent self-rating life events checklist; BDI, beck depression inventory; CTQ, childhood trauma questionnaire; EA, emotional abuse; EN, emotional neglect; HA, health and adaptation problems; IR, interpersonal relationships; L, loss of relatives, friends, and property; LS, learning stress; O, others; P, punishment; PA, physical abuse; PN, physical neglect; SA, sexual abuse; T, total score.

*p < 0.05.

** *p* < 0.01.

## 4 Discussion

We applied the ReHo and rs-FC methods to examine regional activity and distant connectivity, respectively, and found increased ReHo in the ROL.R and decreased FC between the ROL.R, ORBmid.R, and PoCG.L in adolescents with MDD. The abnormal regional ReHo was positively correlated with depression severity and adverse life events, while the abnormal whole-brain FC was negatively correlated with depression severity and adverse experience.

The ROL belongs to the auditory network, which is responsible for integrating and processing auditory stimuli and is associated with human emotions (18). Previous studies have shown that the ROL is involved in interoceptive awareness, which processes signals important for self-awareness (19). In this study, we observed a ReHo anomaly in the ROL.R and as ReHo values increased, the positive relationships between depression severity and adverse life events, although some studies suggested otherwise. Zhang and colleagues discovered that the amplitude of low-frequency fluctuation (ALFF), which quantifies the intensity of local brain activity in a specific frequency bands, was reduced in the ROL.R of patients with MDD (20). Additionally, both ALFF and ReHo are regional functional activity characteristics (21). This discrepancy in results may be attributed to our focus on MDD in adolescents instead of adults. It may provide new possibilities and valuable insights for investigating unique neuroimaging alterations connected to MDD in adolescents.

The middle frontal gyrus, located in the ventral part of the medial prefrontal cortex, is involved in processing and regulation of emotions from top to bottom automatically or implicitly (22). Guo et al. reported that patients with MDD who had not received antidepressant had a decrease in fractional ALFF in the ORBmid.R (23). Furthermore, college students with MDD showed an increased ALFF ratio in the ORBmid.R after exercising (24). Patients with MDD also showed a decrease in the middle frontal and medial orbitofrontal gyri (25). Our findings support previous literature on MDD by identifying abnormalities in the ORBmid.R. The decreased FC between the ROL.R and ORBmid.R related to emotional dysregulation observed in adolescents with MDD could help explain the underlying neurobiological processes contributing to impaired emotion processing in this disorder.

The postcentral gyrus is important in the sensorimotor system (26) and crucial in emotional processing in patients with MDD (27), bipolar disorder (28), and anxiety (29). Luo et al. indicated that PoCG.L activity is linked to childhood trauma and is responsible for the main effect of childhood trauma on adults with MDD. This supports the idea that the postcentral gyrus is involved in the recognition of internal bodily states, as the generation of emotions requires awareness of external and internal bodily states (30). Another study focusing on suicidal behavior of adolescents with depression pointed that higher levels of suicidality were positively associated with functional connectivity between left precuneus and left postcentral gyri (31). This result further supported that the sensorimotor system may underlie the tendency to internal bodily state with negative emotion and suicidal behavior. However, we found decreased FC between the ROL.R and PoCG.L in the adolescents with depression without suicidal behavior in this study. These inconsistent results in the PoCG.L might be due to the differences in the age of participants and the focus of the study. Therefore, we should expand the sample of adolescents with depression and further classify them for analysis according to specific criteria such as whether they have suicidal behavior or whether they have experienced childhood trauma in further studies.

Strong evidence suggests that adverse life events in childhood are the primary risk factors for MDD, as they can affect the ability to recognize and communicate emotions and result in increased psychological distress among adolescents (32, 33). It may be due to an imbalance in the development of subcortical areas (accumbens and amygdala) and prefrontal lobes during the adolescence, therefore, when confronted with negative emotional stimulation, adolescents show less activity in the orbital frontal cortex and more activity in the amygdala than adults (34). The neurological basis for this difference may be a series of prefrontal cortex remodeling that occurs during adolescence, including a decrease in the density of spines on pyramidal cells, a reduction in the volumes and a substantial loss of glutamatergic excitatory synapses (35). Preclinical studies also support the idea that adverse life events have long-lasting effects on neurotransmitters and neurohormonal systems, as well as on a network of brain areas involved in memory and fear, such as the hippocampus, medial prefrontal cortex, and amygdala. These regions interact with neurochemical systems to influence the brain’s response to stress (36). For example, during adolescent development, appropriate thyroid hormone levels could stimulate the differentiation of dopaminergic neurons. However, after adolescence mice experienced early life stress, the thyroid hormone signal transduction development was defective, which further affected the development of ventral tegmental area (37). Other hormones, such as testosterone and estrogen, could also affect the neurotransmission of dopamine, which in turn further impact the maturation of neural circuitry and cognitive processing in adolescence (38). This may be one of the reasons for a large number of female suffering from depression among adolescents, suggesting that the pathological mechanism of hormonal changes related to stress environmental sensitivity in depression should be considered (39).

Despite finding some evidence indicating changes in the neural reactivity of adolescents with MDD who have experienced negative life events and trauma, our study has some limitations. First, as the insufficient sample size restricts the ability to draw precise conclusions, caution is needed when interpreting the findings. Second, the case-control design of this study could only initially explore the correlation between adverse life events and brain function with depression severity in adolescents with depression. In future study, it is necessary to expand the sample size for further cohort studies to explore whether there are changes in brain function and depression symptoms in adolescents with depression after receiving antidepressant medication or cognitive behavioral therapy based on the findings derived from the present study.

In summary, this study expands on the existing literature and emphasizes the critical neurological effects of the cumulative experience of stress and trauma. Additionally, the severity of MDD was found to interact with abnormal activities between the ROL.R and ORBmid.R, and exposure to life stress and trauma was shown to notably influence the PoCG.L and ORBmid.R. These findings provide evidence that there might be distinct developmental patterns of changes in how emotions are processed in the brain, influenced by early adverse life events in adolescents with MDD. Therefore, this study could potentially contribute to research on psychiatric problems during adolescence and necessitate early detection, attention, and individualized intervention to prevent adverse outcomes.

## Data availability statement

The raw data supporting the conclusions of this article will be made available by the authors, without undue reservation.

## Ethics statement

The studies involving humans were approved by Medical Research Ethics Committee of the First Affiliated Hospital of Chongqing Medical University (Ethical approval number: 2023-058-02). The studies were conducted in accordance with the local legislation and institutional requirements. Written informed consent for participation in this study was provided by the participants’ legal guardians/next of kin.

## Author contributions

XX: Formal Analysis, Methodology, Visualization, Writing – original draft, Writing – review & editing. JT: Investigation, Writing – review & editing. YP: Investigation, Writing – review & editing. YL: Investigation, Writing – review & editing. YC: Investigation, Writing – review & editing. MY: Investigation, Writing – review & editing. RY: Data curation, Writing – review & editing. XH: Resources, Writing – review & editing. YF: Conceptualization, Funding acquisition, Project administration, Resources, Supervision, Writing – review & editing.
